# ACCEPTABILITY AND FEASIBILITY OF WEARABLE SENSORS IN AN AGING INDIVIDUAL LIVING COMMUNITY

**DOI:** 10.1093/geroni/igae098.3263

**Published:** 2024-12-31

**Authors:** Rebecca Koszalinski, Ruth Tappen, Behnaz Ghoraani, Edgar Vieira, Borko Furht, David Newman, Muhammad Tanveer Jan

**Affiliations:** University of Central Florida, Greenacres, Florida, United States; Florida Atlantic University, Boca Raton, Florida, United States; Florida Atlantic University, Boca Raton, Florida, United States; Florida International University, Miami, Florida, United States; Florida Atlantic University, Boca Raton, Florida, United States; Florida Atlantic University, Boca Raton, Florida, United States; Florida Atlantic University, Boca Raton, Florida, United States

## Abstract

The use of health technology to detect and measure outcomes in aging individuals offers safe, efficient, and cost-effective detection of changes. There are numerous barriers to acceptability and engagement of technology including participant lack of knowledge, insufficient support, and challenging functionality. It is unclear if barriers of the use of smartwatches stem from participants’ unfamiliarity, inability to use technology, disinterest, or if bias contributes to inadequate engagement and testing with older adults. This team of researchers designed a pilot study to investigate the acceptability of smartwatches among aging individuals residing in adult living community in the Southeastern United States. Out of 27 participants who consented to the study, 7 (25.9%) were requested repeats and all (100%) wore the smartwatches for a period of 1 to 5 days. Engagement was high throughout the data collection period (May – December, 2023), with participants expressing interest in the smartwatches’ data output. Verbatim comments were predominantly positive (“like the look”; “like the technology”; “comfortable”). However, concerns were raised about smartwatch’s size (n = 2), weight (n = 1), and the necessity of maintaining the correct time on the device to facilitate independent time management (n = 5). This study contributes to the literature by highlighting the successful engagement and deliberate inclusion of aging individuals in health technology research, and the acceptability of technology (smartwatches) in this population. It suggests the importance of consulting aging individuals in the planning stages of deploying specific sensor technologies.

